# Presence of the Cyanotoxin Microcystin in Arctic Lakes of Southwestern Greenland

**DOI:** 10.3390/toxins8090256

**Published:** 2016-08-31

**Authors:** Jessica V. Trout-Haney, Zachary T. Wood, Kathryn L. Cottingham

**Affiliations:** 1 Graduate Program in Ecology, Evolution, Ecosystems and Society, Dartmouth College, Hanover, NH 03755, USA; kathryn.cottingham@dartmouth.edu; 2 Department of Biological Sciences, Dartmouth College, Hanover, NH 03755, USA

**Keywords:** cyanotoxins, microcystins, aquatic ecosystems, Arctic, Greenland

## Abstract

Cyanobacteria and their toxins have received significant attention in eutrophic temperate and tropical systems where conspicuous blooms of certain planktonic taxa release toxins into fresh water, threatening its potability and safe use for recreation. Although toxigenic cyanobacteria are not confined to high nutrient environments, bloom-forming species, or planktonic taxa, these other situations are studied les often studied. For example, toxin production in picoplankton and benthic cyanobacteria—the predominant photoautotrophs found in polar lakes—is poorly understood. We quantified the occurrence of microcystin (MC, a hepatotoxic cyanotoxin) across 18 Arctic lakes in southwestern Greenland. All of the focal lakes contained detectable levels of MC, with concentrations ranging from 5 ng·L^−1^ to >400 ng·L^−1^ during summer, 2013–2015. These concentrations are orders of magnitude lower than many eutrophic systems, yet the median lake MC concentration in Greenland (57 ng·L^−1^) was still 6.5 times higher than the median summer MC toxicity observed across 50 New Hampshire lakes between 1998 and 2008 (8.7 ng·L^−1^). The presence of cyanotoxins in these Greenlandic lakes demonstrates that high latitude lakes can support toxigenic cyanobacteria, and suggests that we may be underestimating the potential for these systems to develop high levels of cyanotoxins in the future.

## 1. Introduction

Pelagic blooms of cyanobacteria are of increasing interest worldwide due to their ecological, economic, and public health consequences [1,2]. Cyanotoxins associated with bloom events can cause adverse health effects in humans, domesticated animals, and terrestrial and aquatic organisms [3]. Although cyanotoxin production occurs worldwide, studies of cyanotoxins have traditionally focused on conspicuous blooms of planktonic taxa in nutrient-rich temperate or tropical systems [1,4,5]. However, toxigenic cyanobacteria are not confined to high nutrient environments or planktonic taxa [6,7,8,9,10,11,12,13,14].

Cyanobacteria are often the predominant photoautotrophs in polar freshwater ecosystems [15,16] and can play a major ecological role in these environments [17,18,19]. However, conspicuous pelagic cyanobacterial blooms are extremely rare in these systems as physiological constraints imposed by persistent low temperatures, limited nutrient supply, and high UV-exposure tend to promote growth of benthic and picoplanktonic taxa [15,16]. By comparison to the dozens of studies in lower latitudes (e.g., [1,5,20,21,22,23,24]), relatively few studies have reported the occurrence of cyanotoxins in polar environments [5], with some in the Antarctic [7,8,9,11,13] and even fewer specifically in the Arctic [6,9,10].

Microcystins (MCs) are among the cyanotoxins most widely produced by cyanobacteria genera worldwide [1,25]. MCs act by inhibiting protein phosphatase activity, promoting tumor growth, and inducing·Liver hemorrhaginge [1]. Here we test for the presence of the hepatotoxin microcystin (MC) in whole lake water samples from 18 Arctic lakes located in southwestern Greenland.

## 2. Results

**Figure 1:**
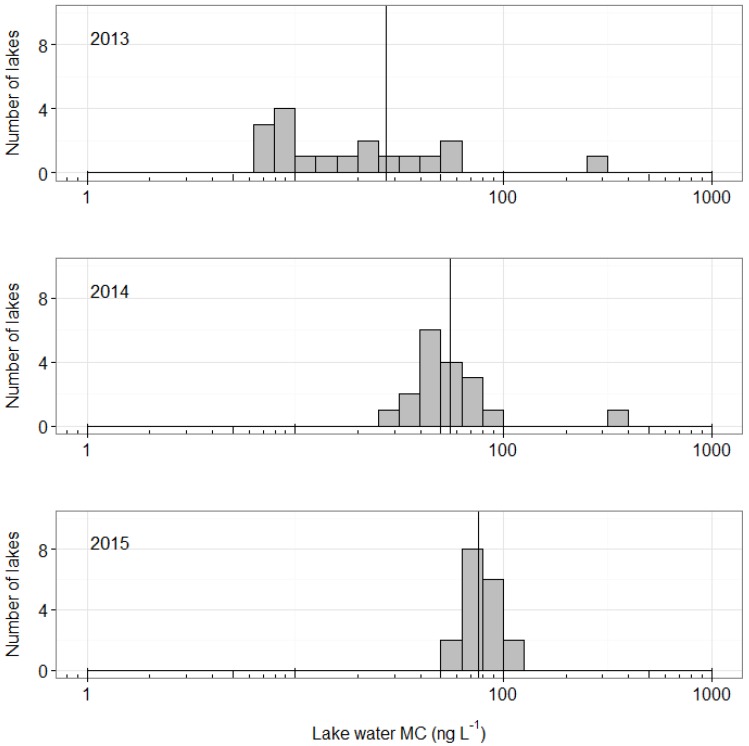
Concentrations of microcystin (MC) ranging from 6 ng·L^−1^ to >300 ng·L^−1^ in 18 lakes in the Kangerlussuaq region of Greenland during the summer, 2013–2015. Vertical lines represent median MC concentrations in each year.

All 18 of the sampled lakes near Kangerlussuaq, Greenland contained detectable levels of MC in “grab” samples of water from the top 1 m, in each of the three years of sampling (2013, 2014, and 2015, Figure 1). Across all lakes, MC concentrations ranged from 6 ng·L^−1^ to >300 ng·L^−1^ (Figure 1), with a median MC concentration of 57 ng·L^−1^ across all lakes and years (Table S1). Excluding the one lake in our data set with very high MC concentrations (lake “SMA”, Table S1), median MC concentrations increased from 14.6 ng·L^−1^ in 2013 to 50.1 ng·L^−1^ in 2014 and 78.9 ng·L^−1^ in 2015 (one-way ANOVA on year controlling for lake as a random effect: F_2,48_ = 55.2, *p* < 0.0001, Figure S1). In quality control (QC) samples, the intra-assay coefficient of variability (CV) was 14% (*n* = 82 plates), while inter-assay CV was 32% (*n* = 21 samples run on ≥2 plates, Table S2).

## 3. Discussion

This study demonstrates that water samples from Greenlandic lakes in the Kangerlussuaq region contain measureable levels of the hepatotoxic cyanotoxin MC. Hence, the dearth of published studies documenting cyanotoxins in polar freshwater habitats may reflect a scarcity in cyanotoxin research in polar regions, rather than their scarcity in these environments.

Importantly, the MC measured in these samples represents toxin that is either free and unbound in the upper 1 m of lake water, or bound-up in plankton biomass and released during the freeze-thaw process—referred to by the World Health Organization as free plus cell-bound MC [24]. As such, our MC values may underestimate the true exposure to MC for organisms within the lake by not explicitly accounting for benthic cyanotoxin pools. For example, Wood et al. [14] detected release of low levels of the cyanotoxin nodularin (which is structurally similar to microcystin) into the water column by benthic periphyton mats. Thus, benthic species could be a source of toxins via both the release of unbound toxin into the water column and any direct consumption of these organisms and/or their epiphytes by higher trophic levels. This issue could be of particular relevance to the Kangerlussuaq region, as many of the studied lakes contain abundant benthic colonial *Nostoc* spp. It is therefore imperative for follow-up studies in this region to quantify the contributions of benthic producers such as *Nostoc* to water column cyanotoxin concentrations in order to understand sources of cyanotoxin in these lakes.

This study corroborates previous work documenting the presence of cyanotoxins in polar environments [6,7,8,9,10,11,13]. However, previous studies have exclusively presented MC concentrations in the context of benthic biological material, such as microbial mats and biological crusts. Therefore, we do not yet know how the whole lake water concentrations measured in this study compare to other polar aquatic environments. Notably, concentrations of MC in surface waters of these Greenlandic lakes are orders of magnitude lower than in many eutrophic temperate or tropical systems that experience extensive pelagic blooms and reach MC concentrations of up to 1.8 mg·L^−1^ [1,12,22]. Indeed, MC concentrations in our study lakes are about an order of magnitude below the median whole lake water MC reported in a nation-wide survey of U.S. lakes (490 ng·L^−1^ free plus cell-bound MC, [22]), and two orders of magnitude below the World Health Organization’s limit for safe human consumption, 1000 ng·L^−1^ [24]. However, substantial regional variation exists and median whole lake water MC in Greenland (57 ng·L^−1^) was 6.5 times higher than the median summer MC concentration observed across 50 New Hampshire lakes between 1998 and 2008 (8.7 ng·L^−1^, [21]).

This study is one of the first cyanotoxin studies to explicitly report the results of QC sample analyses when using ELISA (enzyme-linked immunosorbent assay) techniques (e.g., not reported in [9,10,11,13,20,21,22,23]). While we found that intra-assay precision was relatively high, inter-assay precision was considerably lower. Were it more widely reported, this simple metric could be useful in comparison of both measurements and methodologies across studies. Reporting assay statistics can lend important insight into the precision of the techniques used, and consequently, the interpretation of results. Taking our moderate levels of inter-assay variability into account allows us to better evaluate what constitutes a meaningful difference in sample concentrations and what falls within assay error.

Finally, we observed an increase in median concentrations of MC in these lakes from 2013 to 2015 (Figure 1). While we do not know whether this pattern is representative of a longer-term trend, there may be cause for concern in that Arctic regions are experiencing the fastest and most pronounced impacts of climate change [26]. Projections suggest that West Greenland will continue to see increases in temperature and the size and frequency of precipitation events in the future [27]—changes that may promote the growth of cyanobacterial populations [2,28]. Arctic lakes such as these may therefore be susceptible to shifts in the cyanobacterial community, and with that, the type and amount of toxins produced.

In conclusion, the presence of cyanotoxins in these Greenlandic lakes demonstrates that high-latitude lakes can support toxigenic cyanobacteria and that we may be underestimating the potential for these systems to develop high levels of toxicity in the future.

## 4. Materials and Methods

We tested for MC in whole lake water samples to evaluate the potential presence of free plus cell-bound MC in 18 lakes located in the Kangerlussuaq region of southwestern Greenland (67°01′ N, 50°41′ W). These lakes are situated along a ~30 km transect extending from the head of the *Søndre Strømfjord* and the margin of the Greenland Ice Sheet, and include small kettle ponds, shallow cryogenic lakes, large cirque lakes, and deep fault-valley lakes (Table S1). In June and July of 2013–2015, we collected and combined three subsamples of water (250–1000 mL) from the upper 1 m of each lake to represent one composite sample per lake. All water samples were immediately frozen (−20 °C) until laboratory analyses.

In analyzing·Lake water MC concentrations we followed extraction protocols adapted from [18]. MC was detected using the high sensitivity protocol for an enzyme-linked immunosorbent assay (ELISA) with a method limit of detection (LOD) of 30 ng·L^−1^ (Envirologix, Inc., Portland, ME, USA). The ELISA kit does not distinguish between microcystin variants and as such we use the term MC to refer to four possible microcystin toxin variants (MC-LR, MC-LA, MC-RR, MC-YR) and the structurally similar nodularin toxin. Prior to the ELISA, the entirety of each water sample was subjected to a toxin extraction process consisting of triplicate cycles of freeze-thaw (−80 °C, minimum freeze time of 1 h) and incubation in a sonic water bath (5–10 min intervals). Samples were centrifuged and the supernatants collected for analysis. When extracted samples were below the method detection limit, water was transferred to borosilicate serum bottles, refrozen, and lyophilized in a freeze-dry system (Labconco) under vacuum (~30 × 10^3^ mbar) at −50 °C for 18–24 h. Samples were then rehydrated with distilled water to achieve a 10-fold increase in the original concentration and final measurements were corrected for this concentration factor. We analyzed the change in lake MC concentrations over time using a one-way ANOVA controlling for lake as a random effect in R (version 3.2.0) using the “stats” package [29] *aov()* function and ggplot2 [30] for graphical output.

To evaluate assay precision, we analyzed quality control (QC) samples using randomly selected water samples collected in Greenland (2013–2015). We replicated subsamples within ELISA plates (intra-assay variation) and across plates run on different days (inter-assay variation) to determine the degree of repeatability in measurements of MC concentrations. We calculated intra-assay variation as the standard deviation (SD) of duplicate sample concentrations within a plate divided by the mean concentration of those duplicates × 100, expressed as the % coefficient of variation (% CV). We determined inter-assay variation by calculating the % CV across plate-specific sample means.

## Supplementary Materials

The following are available online at www.mdpi.com/2072-6651/8/9/256/s1, Figure S1: Median concentrations in lake water MC in Greenlandic lakes from 2013 to 2015, Table S1: Physical parameters of Greenlandic study lakes; Table S2: Means, medians, and coefficients of variation for each lake water sample.

## Abbreviations

The following abbreviations are used in this manuscript:

MC: Microcystin, a potent hepatotoxic cyanotoxin produced by a wide range of cyanobacterial taxa and frequently detected. MC refers to four possible microcystin variants (MC-LR, MC-LA, MC-RR, MC-YR) and nodularin toxin (this ELISA does not distinguish between microcystin toxin variants).

## References

[1] Sivonen K., Jones G., Chorus I., Bartram J. (1999). Chapter 3: Cyanobacterial toxins. Toxic Cyanobaceria in Water: A Guide to their Public Health Consequences, Monitoring and Management.

[2] Paerl H.W., Paul V.J. (2012). Climate change: Links to global expansion of harmful cyanobacteria. Water Res..

[3] Hilborn E.D., Beasley V.R. (2015). One health and cyanobacteria in freshwater systems: Animal illnesses and deaths are sentinel events for human health risks. Toxins.

[4] Sinha R., Pearson L.A., Davis T.W., Burford M.A., Orr P.T., Neilan B.A. (2012). Increased incidence of *Cylindrospermopsis raciborskii* in temperate zones—Is climate change responsible?. Water Res..

[5] Metcalf J.S., Codd G.A. (2014). Cyanobacterial toxins (cyanotoxins) in water. Found. Water Res..

[6] Chrapusta E., Węgrzyn M., Zabaglo K., Kaminski A., Adamski M., Wietrzyk P., Bialczyk J. (2015). Microcystins and anatoxin-a in Arctic biocrust cyanobacterial communities. Toxicon.

[7] Hitzfeld B.C., Lampert C.S., Spaeth N., Mountfort D., Kaspar H., Dietrich D.R. (2000). Toxin production in cyanobacterial mats from ponds on the McMurdo Ice Shelf, Antarctica. Toxicon.

[8] Jungblut A.-D., Hoeger S.J., Mountfort D., Hitzfeld B.C., Dietrich D.R., Neilan B.A. (2006). Characterization of microcystin production in an Antarctic cyanobacterial mat community. Toxicon.

[9] Kleinteich J., Wood S.A., Küpper F.C., Camacho A., Quesada A., Frickey T., Dietrich D.R. (2012). Temperature-related changes in polar cyanobacterial mat diversity and toxin production. Nat. Clim. Chang..

[10] Kleinteich J., Wood S.A., Puddick J., Schleheck D., Küpper F.C., Dietrich D. (2013). Potent toxins in Arctic environments—Presence of saxitoxins and an unusual microcystin variant in Arctic freshwater ecosystems. Chem. Biol. Interact..

[11] Kleinteich J., Hildebrand F., Wood S.A., Cirés S., Agha R., Quesada A., Pearce D.A., Convey P., Küpper F.C., Dietrich D.R. (2014). Diversity of toxin and non-toxin containing cyanobacterial mats of meltwater ponds on the Antarctic Peninsula: A pyrosequencing approach. Antarct. Sci..

[12] Quiblier C., Wood S., Echenique-Subiabre I., Heath M., Villeneuve A., Humbert J.-F. (2013). A review of current knowledge on toxic benthic freshwater cyanobacteria – Ecology, toxin production and risk management. Water Res..

[13] Wood S.A., Mountfort D., Selwood A.I., Holland P.T., Puddick J., Cary S.C. (2008). Widespread distribution and identification of eight novel microcystins in Antarctic cyanobacterial mats. Appl. Environ. Microbiol..

[14] Wood S.A., Kuhajek J.M., de Winton M., Phillips N.R. (2012). Species composition and cyanotoxin production in periphyton mats from three lakes of varying trophic status. FEMS Microbiol. Ecol..

[15] Callieri C., Cronberg G., Stockner J., Whitton B.A. (2012). Chapter 8: Freshwater picocyanobacteria: Single cells, microcolonies, and colonial forms. Ecology of Cyanobacteria II: Their Diversity in Space and Time.

[16] Vincent W.R., Quesada A., Whitton B.A. (2012). Chapter 13: Cyanobacteria in high latitude lakes, rivers, and seas. Ecology of Cyanobacteria II: Their Diversity in Space and Time.

[17] Prepas E.E., Kotak B.G. (1997). Accumulation and elimination of cyanobacterial hepatotoxins by the freshwater clam *Anodonta grandis simpsoniana*. Can. J. Fish. Aquat. Sci..

[18] Tang E.P.Y., Tremblay R., Vincent W.F. (1997). Cyanobacterial dominance of polar freshwater ecosystems. J. Phycol..

[19] Vincent W.F., Hobbie J.E., Nuttall M., Callaghan T. (2000). Ecology of Arctic Lakes. The Arctic: Environment, People, Policy.

[20] Frank C.A.P. (2002). Microcystin-producing cyanobacteria in recreational waters in southwestern Germany. Environ. Toxicol..

[21] Haney J., Ikawa M. (2000). A survey of 50 NH lakes for microcystins (MCs). UNH Cent. Freshw. Biol. Res..

[22] Loftin K.A., Graham J.L., Hilborn E.D., Lehmann S.C., Meyer M.T., Dietze J.E., Griffith C.B. (2016). Cyanotoxins in inland lakes of the United States: Occurrence and potential recreational health risks in the EPA National Lakes Assessment 2007. Harmful Algae.

[23] Ueno Y., Nagata S., Tsutsumi T., Hasegawa A., Yoshida F., Suttajit M., Mebs D., Pütsch M., Vasconcelos V. (1996). Survey of microcystins in environmental water by a highly sensitive immunoassay based on monoclonal antibody. Nat. Toxins.

[24] Sheffer M., World Health Organization Guidelines for Drinking-water Quality (2011). Guidelines for Drinking Water Quality.

[25] Carmichael W.W. (1992). Cyanobacteria secondary metabolites—The cyanotoxins. J. Appl. Bacteriol..

[26] Pachauri R.K., Meyer L.A., Intergovernmental Panel on Climate Change (IPCC) (2014). Contributions of Working Groups I, II and III to the Fifth Assessment Report of the Intergovernmental Panel on Climate Change. Climate Change 2014: Synthesis Report.

[27] Stendel M., Christensen J.H., Petersen D. (2008). High-Arctic ecosystem dynamics in a changing climate. Advances in Ecological Research.

[28] Paerl H.W., Huisman J. (2008). Climate. Blooms like it hot. Science.

[29] R Core Team (2015). R: A Language and Environment for Statistical Computing.

[30] Wickham H. (2009). ggplot2: Elegant Graphics for Data Analysis.

