# Supplementary material for: Presence of the Cyanotoxin Microcystin in Arctic Lakes of Southwestern Greenland
Source: Toxins (Basel). 2016 Aug 31;8(9):256. doi: 10.3390/toxins8090256 (PMC5037482; doi:10.3390/toxins8090256)
Supplement: Supplementary file 1 [file toxins-08-00256-s001.pdf]

Supplementary Materials: Presence of the Cyanotoxin Microcystin in Arctic Lakes of Southwestern Greenland

Jessica V. Trout-Haney, Zachary T. Wood and Kathryn L. Cottingham

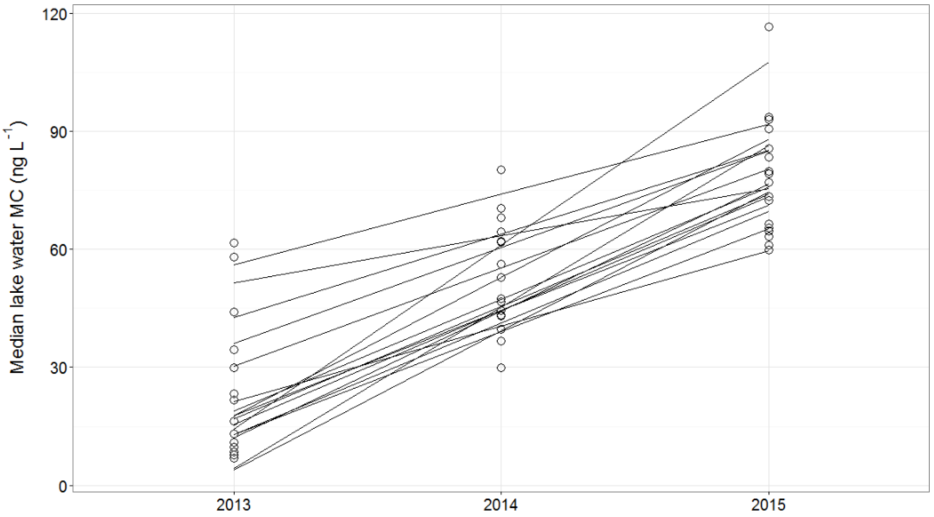

Figure S1. Median lake water microcystin (MC) for 18 Greenlandic lakes from June and July of 2013–2015.

Table S1. Physical parameters in 18 lakes located in Kangerlussauq, Greenland from June and July of 2013–2015.

Lake Code	Latitude (N)	Longitude (W)	Max Depth (m)	Surface Area (ha)
BCL	67.05738	50.4404	2.5	7860.5
BL	67.14352	50.0893	1	808
BSL	67.04852	50.5199	2.8	4549
EIP	67.14808	50.0801	3.5	700.6
HL	67.04823	50.4958	5.3	307.6
LCL	67.05652	50.4500	4.8	1407
LH	66.98683	50.9219	4.8	37,926.4
LSL	67.04870	50.5186	1.8	1756.7
LWL	67.08730	50.2864	5.8	172.5
ML	67.12972	50.1737	11	37,662.4
NBL	67.00055	50.8058	7.5	654.5
PL	67.14480	50.0806	1	1887.5
SBL	67.00037	50.8059	6	657.4
SMA	67.03977	50.5603	12	9479.5
SMI	67.03893	50.5668	4	2719.3
STL	67.05608	50.4645	10	15,319.9
TSL	67.04815	50.5076	1.8	598
WL	67.08688	50.2924	2.8	1263.4

Table S2. Mean and median concentrations of free and cell-bound microcystin (MC, ng L^{-1}) in water from 18 lakes in Kangerlussuaq, Greenland from 2013 to 2015. Intra-assay % CV (coefficient of variability) reflects variation within a plate, and inter-assay % CV reflects variation across plates (calculated for samples run on ≥2 plates). NA: not available.

Lake Code	Year	Total N	Median Across Plates	Mean Across Plates	Intra-Assay %CV	Inter-Assay %CV	Total Plates
BCL	2013	4	10.9	10.8	5.7	7.4	2
BCL	2014	2	43.0	43.0	18.6	NA	1
BCL	2015	2	63.3	63.3	8.1	NA	1
BL	2013	2	23.3	23.3	29.8	NA	1
BL	2014	2	43.1	43.1	10.1	NA	1
BL	2015	2	116.7	116.7	3.0	NA	1
BSL	2013	4	8.5	9.0	10.5	10.3	2
BSL	2014	6	68.0	66.3	17.1	29.2	3
BSL	2015	4	65.6	65.0	6.3	15.6	2
EIP	2013	2	6.8	6.8	9.5	NA	1
EIP	2014	4	62.0	60.7	10.8	4.1	2
EIP	2015	2	64.8	64.8	15.5	NA	1
HL	2013	2	16.2	16.2	19.6	NA	1
HL	2014	2	46.7	46.7	44.8	NA	1
HL	2015	2	73.4	73.4	2.9	NA	1
LCL	2013	4	8.6	9.5	10.1	68.2	2
LCL	2014	2	36.6	36.6	6.2	NA	1
LCL	2015	2	90.8	90.8	7.7	NA	1
LH	2013	2	7.6	7.6	20.0	NA	1
LH	2014	4	64.5	65.0	30.3	2.0	2
LH	2015	2	61.2	61.2	11.2	NA	1
LSL	2013	2	44.2	44.2	8.5	NA	1
LSL	2014	2	44.6	44.6	12.3	NA	1
LSL	2015	2	93.0	93.0	6.6	NA	1
LWL	2013	6	61.7	58.6	17.3	65.3	3
LWL	2014	2	43.3	43.3	27.4	NA	1
LWL	2015	4	85.7	86.2	5.8	30.6	2
ML	2013	2	7.7	7.7	41.6	NA	1
ML	2014	4	53.0	52.7	12.9	40.6	2
ML	2015	4	72.4	73.1	1.7	4.9	2
NBL	2013	4	9.7	9.4	18.2	13.8	2
NBL	2014	2	47.5	47.5	4.9	NA	1
NBL	2015	2	66.5	66.5	13.6	NA	1
PL	2013	2	8.6	8.6	27.5	NA	1
PL	2014	2	29.9	29.9	6.0	NA	1
PL	2015	2	79.3	79.3	36.2	NA	1
SBL	2013	4	21.8	24.6	20.9	89.4	2
SBL	2014	2	39.7	39.7	0.1	NA	1
SBL	2015	2	60.0	60.0	10.6	NA	1
SMA	2013	6	177.6	291.1	20.6	77.7	3
SMA	2014	6	382.1	317.4	6.1	45.9	3
SMA	2015	4	115.9	116.2	1.5	21.0	2
SMI	2013	2	58.0	58.0	2.0	NA	1
SMI	2014	2	70.4	70.4	16.7	NA	1
SMI	2015	1	93.7	93.7	NA	NA	1
STL	2013	6	34.4	37.8	9.1	70.7	3
STL	2014	6	80.4	79.5	5.5	4.8	3
STL	2015	4	77.1	78.4	9.4	39.2	2
TSL	2013	2	29.9	29.9	127.6	NA	1
TSL	2014	2	56.2	56.2	6.9	NA	1
TSL	2015	2	80.0	80.0	0.2	NA	1
WL	2013	2	13.1	13.1	54.1	NA	1
WL	2014	6	62.2	54.8	9.6	32.6	3
WL	2015	4	83.4	80.7	6.3	6.9	2
